# Drugs Used in the Treatment of Rheumatoid Arthritis: Relationship between Current Use and Cardiovascular Risk Factors

**DOI:** 10.1111/j.1753-5174.2009.00019.x

**Published:** 2009-06

**Authors:** Young Hee Rho, Annette Oeser, Cecilia P Chung, Ginger L Milne, C Michael Stein

**Affiliations:** Divisions of Clinical Pharmacology and Rheumatology, Vanderbilt University School of MedicineNashville, TN, USA

**Keywords:** Corticosteroids, Methotrexate, Antimalarials, NSAIDs, COX-2 Inhibitor, Leflunomide, Rheumatoid arthritis, Cardiovascular Risk

## Abstract

**Objectives:**

Drugs used for the treatment of rheumatoid arthritis (RA) have the potential to affect cardiovascular risk factors. There is concern that corticosteroids, non-steroidal anti-inflammatory drugs (NSAIDs) and COX-2 inhibitors could affect cardiovascular risk adversely, while drugs such as the antimalarial, hydroxychloroquine, may have beneficial effects. However, there is limited information about cardiovascular risk factors in patients with RA receiving different drugs.

**Methods:**

We measured cardiovascular risk factors including systolic and diastolic blood pressure, serum HDL and LDL cholesterol, glucose and homocysteine concentrations and urinary F_2_-isoprostane excretion in 169 patients with RA. Risk factors were compared according to current use of corticosteroids, methotrexate, antimalarials, NSAIDs, COX-2 inhibitors, leflunomide and TNF-α blockers. Comparisons were adjusted for age, sex, race, disease activity (DAS28 score), current hypertension, diabetes, smoking status and statin use.

**Results:**

No cardiovascular risk factor differed significantly among current users and non-users of NSAIDs, COX-2 inhibitors, methotrexate and TNF-α blockers. Serum HDL cholesterol concentrations were significantly higher in patients currently receiving corticosteroids (42.2 ± 10.5 vs. 50.2 ± 15.3 mg/dL, adjusted *P* < 0.001). Diastolic blood pressure (75.9 ± 11.2 vs. 72.0 ± 9.1 mm Hg, adjusted *P* = 0.02), serum LDL cholesterol (115.6 ± 34.7 vs. 103.7 ± 27.8 mg/dL, adjusted *P* = 0.03) and triglyceride concentrations (157.7 ± 202.6 vs. 105.5 ± 50.5 mg/dL, adjusted *P* = 0.03) were significantly lower in patients taking antimalarial drugs. Plasma glucose was significantly lower in current lefunomide users (93.0 ± 19.2 vs. 83.6 ± 13.4 mg/dL, adjusted *P* = 0.006).

**Conclusions:**

In a cross-sectional setting drugs used to treat RA did not have major adverse effects on cardiovascular risk factors and use of antimalarials was associated with beneficial lipid profiles.

## Introduction

Coronary atherosclerosis [1] and cardiovascular mortality [2] are increased in patients with rheumatoid arthritis (RA). These patients are treated with a range of drugs including non-steroidal anti-inflammatory drugs (NSAIDs), selective cyclo-oxygenase-2 (COX-2) inhibitors, corticosteroids and disease modifying anti-rheumatic drugs (DMARDs) including antimalarials, methotrexate, tumor necrosis factor alpha (TNF-α) blockers and leflunomide. Drugs used to treat RA may affect cardiovascular outcomes. For example, in large epidemiological studies DMARDs such as methotrexate appear to decrease cardiovascular mortality [3,4], while COX-2 selective drugs increase it [5,6]. Thus, it is important to understand the effects of drugs used to treat RA on cardiovascular risk factors.

There is some information about the effects of drugs used to treat RA on risk factors. For example, NSAIDs and COX-2 inhibitors increase blood pressure [7], high doses of corticosteroids can cause diabetes [8], and antimalarials may improve glucose tolerance [9]. However, the information available is limited. We examined the hypothesis that drugs used to treat RA affected common cardiovascular risk factors.

## Methods

### Patients and Control Subjects

One hundred and sixty nine (169) patients with RA were recruited as previously described [1]. Patients were older than 18 years, fulfilled the ACR classification criteria for RA, and are participating in ongoing studies of cardiovascular risk in RA [10–12]. The study was approved by the Vanderbilt University Institutional Review Board and subjects gave written informed consent.

### Clinical Measurements and Indices/Scores

Clinical information and laboratory data were obtained as described [1]. Hypertension was defined as systolic blood pressure ≥140 mm Hg, or diastolic blood pressure ≥90 mm Hg at enrollment, or currently receiving antihypertensive treatment. Diabetes was defined as a fasting blood glucose concentration >126 mg/dL at enrollment or currently receiving anti-diabetic treatment. The disease activity score (DAS28) [13] includes 4 measures: a 28 swollen joint count, 28 tender joint count, the erythrocyte sedimentation rate (ESR), and patient global estimate of disease status on a 10 cm visual analog scale (VAS). Patients were classified according to whether they were currently taking or not taking the following drugs: corticosteroids, methotrexate, antimalarials, NSAID, COX-2, leflunomide and anti-TNF blockers. In patients not currently taking these drugs we recorded whether they were past users. Fasting plasma glucose and homocysteine and serum triglyceride, HDL and LDL cholesterol concentrations were measured at the Vanderbilt University Medical Center Clinical Laboratory facilities. Urinary F_2_-isoprostanes, a measure of oxidative stress that has been associated with cardiovascular risk in other studies [14], were quantified using gas chromatography and mass spectroscopy [15] and expressed as ng/mg creatinine (ng/mg Cr).

### Statistical Analysis

Descriptive statistics were calculated as mean with standard deviation (SD) according to distributions of continuous variables. Differences in cardiovascular risk factors such as systolic blood pressure (SBP), diastolic blood pressure (DBP), concentrations of serum HDL, LDL, and triglycerides, plasma glucose and homocysteine, and urinary F_2_-isoprostane excretion were compared in patients using or not currently using each drug using the Wilcoxon rank-sum test. The difference was further adjusted for age, sex, race, DAS28 score, current hypertension, diabetes, smoking status and statin use by a multiple linear regression model. The inclusion of hypertension and diabetes status as covariates was to account for use of antihypertensive and hypoglycemic agents. Similarly, adjusting for statin use controlled for differences in lipids accounted for by therapy. Our goal was to adjust for confounding as much as possible since cardiovascular risk is affected by the factors considered and inflammation [11,16]. Thus, while the results may be conservative they will also be robust. To exclude the potential confounding effects of co-therapies we constructed additional statistical models for each drug that, in addition to the variables previously included, also adjusted for the other drugs. Serum triglycerides and urinary F_2_-isoprostanes were log-transformed due to their skewed distribution. Statistical significance was determined using 2-sided 5% significance level (i.e., *P* value <0.05). Statistical analysis was performed using R 2.7.2 (http://www.r-project.org).

## Results

The characteristics of the patients are shown in Table 1. The patients were predominantly middle-aged and female, reflecting the epidemiology of RA. The differences in cardiovascular risk factors between current users and non-user of each particular class of drug are shown in Tables 2 to 8. No cardiovascular risk factor differed significantly among current users and non-users of NSAIDs, COX-2 inhibitors, methotrexate and TNF-α blockers.

**Table 1:** Descriptive statistics of the study group (N = 169)

Factor	Mean ± SD or percentage
Sex (males)	30.8%
Age (years)	54.2 ± 11.8
Race (Caucasians)	88.2%
DAS28 score	3.79 ± 1.61
Hypertension	53.3%
Diabetes	11.2%
Statin use	12.4%
Systolic blood pressure (mm Hg)	133.3 ± 20.3
Diastolic blood pressure (mm Hg)	74.9 ± 10.8
HDL (mg/dL)	46.6 ± 13.9
LDL (mg/dL)	112.7 ± 33.4
Triglycerides (mg/dL)	144.6 ± 178.5
Glucose (mg/dL)	91.2 ± 18.6
Homocysteine (µmol/L)	10.5 ± 3.4
F_2_-Isoprostanes (ng/mg Creatinine)	3.49 ± 3.80
Current corticosteroid use	54.4%
Current methotrexate use	71.0%
Current antimalarial use	24.9%
Current NSAID use	33.1%
Current COX-2 inhibitor use	30.2%
Current leflunomide use	18.3%
Current anti-TNF blocker use	20.7%
Past steroid use	34.3%
Past methotrexate use	20.1%
Past antimalarial use	13.0%
Past NSAID use	18.9%
Past COX-2 inhibitor use	36.7%
Past anti-TNF blocker use	6.5%

**Table 2:** Cardiovascular risk factors and current corticosteroid use

Risk factor	No steroids (N = 77)	Current steroid use (N = 92)	*P* value	Beta* (95%CI)	*P* value*
Systolic BP (mm Hg)	134.1 ± 20.2	132.7 ± 20.5	0.51	1.47 (−3.35–6.29)	0.55
Diastolic BP (mm Hg)	73.2 ± 10.3	76.4 ± 11.1	0.06	2.71 (−0.39–5.82)	0.09
HDL (mg/dL)	42.2 ± 10.5	50.2 ± 15.3	<0.001	9.47 (5.66–13.27)	<0.001
LDL (mg/dL)	115.4 ± 33.0	110.4 ± 33.8	0.44	−5.19 (−15.9–5.53)	0.34
Triglycerides (mg/dL)	146.8 ± 97.2	142.8 ± 225.2	0.004	−0.11 (−0.27–0.06)	0.22
Glucose (mg/dL)	93.8 ± 19.5	89.1 ± 17.5	0.01	−3.86 (−8.45–0.74)	0.10
Homocysteine (µmol/L)	9.90 ± 2.80	11.02 ± 3.70	0.07	0.64 (−0.3–1.58)	0.18
F_2_-Isoprostanes (ng/mg Cr)	3.30 ± 2.53	3.66 ± 4.61	0.75	0.08 (−0.12–0.28)	0.44

* Adjusted for age, sex, race, DAS28, hypertension status, diabetes status, current statin use and current smoking status.

Beta indicates the raw regression coefficient of each drug in the adjusted model.

Note that triglycerides and F_2_-isoprostanes were log-transformed so the coefficients reflect mean differences in log-units than raw units.

**Table 3:** Cardiovascular risk factors and current methotrexate use

Risk factor	No methotrexate (N = 49)	Current methotrexate use (N = 120)	*P* value	Beta* (95%CI)	*P* value*
Systolic BP (mm Hg)	137.5 ± 18.0	131.6 ± 21.0	0.09	−1.35 (−6.67–3.97)	0.62
Diastolic BP (mm Hg)	77.9 ± 9.5	73.7 ± 11.1	0.02	−1.85 (−5.29–1.59)	0.29
HDL (mg/dL)	43.5 ± 11.0	47.8 ± 14.8	0.12	2.89 (−1.62–7.39)	0.21
LDL (mg/dL)	115.0 ± 38.1	111.7 ± 31.4	0.75	−4.15 (−16.04–7.74)	0.50
Triglycerides (mg/dL)	150.0 ± 113.0	142.0 ± 200.0	0.18	−0.11 (−0.3–0.07)	0.24
Glucose (mg/dL)	92.2 ± 21.5	90.9 ± 17.3	0.91	2.91 (−2.19–8.01)	0.27
Homocysteine (µmol/L)	11.49 ± 4.28	10.11 ± 2.82	0.10	−0.47 (−1.51–0.58)	0.38
F_2_-Isoprostanes (ng/mg Cr)	3.31 ± 2.31	3.57 ± 4.27	0.96	−0.10 (−0.32–0.12)	0.36

* Adjusted for age, sex, race, DAS28, hypertension status, diabetes status, current statin use and current smoking status.

Beta indicates the raw regression coefficient of each drug in the adjusted model.

Note that triglycerides and F_2_-isoprostanes were log-transformed so the coefficients mean reflect differences in log-units than raw units.

**Table 4:** Cardiovascular risk factors and current antimalarial use

Risk factor	No antimalarials (N = 127)	Current antimalarial use (N = 42)	*P* value	Beta* (95%CI)	*P* value*
Systolic BP (mm Hg)	135.5 ± 19.8	126.7 ± 20.5	0.01	−4.59 (−9.99–0.82)	0.10
Diastolic BP (mm Hg)	75.9 ± 11.2	72.0 ± 9.1	0.047	−4.04 (−7.52–−0.56)	0.02
HDL (mg/dL)	45.3 ± 13.7	50.4 ± 14.1	0.03	3.94 (−0.62–8.5)	0.09
LDL (mg/dL)	115.6 ± 34.7	103.7 ± 27.8	0.07	−13.37 (−25.29–−1.45)	0.03
Triglycerides (mg/dL)	157.7 ± 202.6	105.5 ± 50.5	0.006	−0.21 (−0.4–−0.02)	0.03
Glucose (mg/dL)	92.2 ± 18.8	88.4 ± 17.7	0.09	−0.01 (−5.26–5.23)	1.00
Homocysteine (µmol/L)	10.56 ± 3.21	10.34 ± 3.78	0.59	0.10 (−0.97–1.16)	0.86
F_2_-Isoprostanes (ng/mg Cr)	3.60 ± 4.23	3.18 ± 2.00	0.87	−0.11 (−0.33–0.12)	0.35

* Adjusted for age, sex, race, DAS28, hypertension status, diabetes status, current statin use and current smoking status.

Beta indicates the raw regression coefficient of each drug in the adjusted model.

Note that triglycerides and F_2_-isoprostanes were log-transformed so the coefficients reflect mean differences in log-units than raw units.

**Table 5:** Cardiovascular risk factors and current NSAID use

Risk factor	No NSAIDs (N = 113)	Current NSAID use (N = 56)	*P* value	Beta* (95%CI)	*P* value*
Systolic BP (mm Hg)	134.5 ± 20.8	130.8 ± 19.3	0.34	−1.03 (−6.12–4.07)	0.69
Diastolic BP (mm Hg)	75.5 ± 10.7	73.8 ± 10.9	0.27	−0.65 (−3.96–2.66)	0.70
HDL (mg/dL)	46.8 ± 13.4	46.1 ± 15.0	0.66	2.33 (−1.96–6.63)	0.29
LDL (mg/dL)	113.7 ± 34.0	110.5 ± 32.3	0.62	−0.85 (−12.2–10.49)	0.88
Triglycerides (mg/dL)	148.7 ± 208.7	136.4 ± 90.4	0.79	−0.03 (−0.21–0.14)	0.71
Glucose (mg/dL)	90.6 ± 16.2	92.6 ± 22.7	0.71	−0.39 (−5.29–4.52)	0.88
Homocysteine (µmol/L)	10.61 ± 3.30	10.29 ± 3.48	0.59	−0.05 (−1.05–0.95)	0.92
F_2_-Isoprostanes (ng/mg Cr)	3.19 ± 2.40	4.11 ± 5.62	0.17	0.11 (−0.1–0.32)	0.31

* Adjusted for age, sex, race, DAS28, hypertension status, diabetes status, current statin use and current smoking status.

Beta indicates the raw regression coefficient of each drug in the adjusted model.

Note that triglycerides and F_2_-isoprostanes were log-transformed so the coefficients reflect mean differences in log-units than raw units.

**Table 6:** Cardiovascular risk factors and current COX-2 inhibitor use

Risk factor	No COX-2 inhibitors (N = 118)	Current COX-2 inhibitor use (N = 51)	*P* value	Beta* (95%CI)	*P* value*
Systolic BP (mm Hg)	133.7 ± 20.5	132.5 ± 19.9	0.60	−1.66 (−6.75–3.44)	0.53
Diastolic BP (mm Hg)	75.2 ± 10.9	74.3 ± 10.6	0.58	−0.9 (−4.21–2.42)	0.60
HDL (mg/dL)	46.1 ± 13.0	47.7 ± 15.8	0.78	0.13 (−4.18–4.44)	0.95
LDL (mg/dL)	114.4 ± 34.3	108.8 ± 31.2	0.33	−8.24 (−19.5–3.03)	0.15
Triglycerides (mg/dL)	151.6 ± 208.1	128.7 ± 75.0	0.93	−0.03 (−0.2–0.15)	0.76
Glucose (mg/dL)	91.9 ± 20.5	89.8 ± 13.0	0.82	0.32 (−4.59–5.22)	0.90
Homocysteine (µmol/L)	10.69 ± 3.50	10.09 ± 2.97	0.32	−0.32 (−1.32–0.67)	0.53
F_2_-Isoprostanes (ng/mg Cr)	3.59 ± 4.17	3.28 ± 2.78	0.44	−0.04 (−0.25–0.17)	0.69

* Adjusted for age, sex, race, DAS28, hypertension status, diabetes status, current statin use and current smoking status.

Beta indicates the raw regression coefficient of each drug in the adjusted model.

Note that triglycerides and F_2_-isoprostanes were log-transformed so the coefficients reflect mean differences in log-units than raw units.

**Table 7:** Cardiovascular risk factors and current leflunomide use

Risk factor	No leflunomide (N = 138)	Current leflunomide use (N = 31)	*P* value	Beta* (95%CI)	*P* value*
Systolic BP (mm Hg)	132.6 ± 20.3	136.6 ± 20.2	0.28	5.7 (−0.32–11.73)	0.07
Diastolic BP (mm Hg)	74.6 ± 10.6	76.5 ± 11.6	0.33	1.41 (−2.54–5.35)	0.49
HDL (mg/dL)	46.4 ± 14.1	47.1 ± 13.4	0.69	0.74 (−4.39–5.88)	0.78
LDL (mg/dL)	111.5 ± 33.3	117.9 ± 34.2	0.28	8.40 (−5.06–21.86)	0.22
Triglycerides (mg/dL)	145.0 ± 187.8	143.0 ± 132.2	0.59	0.02 (−0.19–0.23)	0.86
Glucose (mg/dL)	93.0 ± 19.2	83.6 ± 13.4	0.01	−8.12 (−13.84–−2.41)	0.006
Homocysteine (µmol/L)	10.37 ± 3.20	11.09 ± 3.97	0.68	0.50 (−0.69–1.68)	0.41
F_2_-Isoprostanes (ng/mg Cr)	3.36 ± 4.02	4.07 ± 2.59	0.03	0.19 (−0.06–0.44)	0.15

* Adjusted for age, sex, race, DAS28, hypertension status, diabetes status, current statin use and current smoking status.

Beta indicates the raw regression coefficient of each drug in the adjusted model.

Note that triglycerides and F_2_-isoprostanes were log-transformed so the coefficients reflect mean differences in log-units than raw units.

**Table 8:** Cardiovascular risk factors and current TNF-α blocker use

Risk factor	No TNF blockers (N = 134)	Current TNF blocker use (N = 35)	*P* value	Beta* (95%CI)	*P* value*
Systolic BP (mm Hg)	133.6 ± 21.2	132.2 ± 16.7	0.91	1.73 (−4.08–7.53)	0.56
Diastolic BP (mm Hg)	74.6 ± 10.7	76.3 ± 11.1	0.36	3.47 (−0.26–7.21)	0.07
HDL (mg/dL)	45.6 ± 13.7	50.2 ± 14.2	0.06	2.87 (−2.01–7.75)	0.25
LDL (mg/dL)	111.6 ± 31.1	116.7 ± 41.2	0.92	3.71 (−9.18–16.6)	0.57
Triglycerides (mg/dL)	136.4 ± 96.4	176.1 ± 345.2	0.97	0.03 (−0.17–0.23)	0.76
Glucose (mg/dL)	92.7 ± 19.2	85.7 ± 14.7	0.04	−3.48 (−9.04–2.09)	0.22
Homocysteine (µmol/L)	10.82 ± 3.46	9.29 ± 2.60	0.008	−0.86 (−1.99–0.28)	0.14
F_2_-Isoprostanes (ng/mg Cr)	3.26 ± 2.44	4.42 ± 6.92	0.25	0.18 (−0.07–0.42)	0.15

* Adjusted for age, sex, race, DAS28, hypertension status, diabetes status, current statin use and current smoking status.

Beta indicates the raw regression coefficient of each drug in the adjusted model.

Note that triglycerides and F_2_-isoprostanes were log-transformed so the coefficients reflect mean differences in log-units than raw units.

Current corticosteroid use was associated with higher HDL cholesterol concentrations (42.2 ± 10.5 vs. 50.2 ± 15.3 mg/dL, adjusted *P* < 0.001). Current use of antimalarial therapy (almost exclusively hydroxychloroquine) was associated with lower diastolic blood pressure (75.9 ± 11.2 vs. 72.0 ± 9.1 mm Hg, adjusted *P* = 0.02), serum LDL (115.6 ± 34.7 vs. 103.7 ± 27.8 mg/dL, adjusted *P* = 0.03) and triglyceride concentrations (157.7 ± 202.6 vs. 105.5 ± 50.5 mg/dL, adjusted *P* = 0.03). Plasma glucose was significantly lower in patients taking lefunomide (93.0 ± 19.2 vs. 83.6 ± 13.4 mg/dL, adjusted *P* = 0.006). In a post-hoc analysis, current leflunomide use was associated with a marginally lower body mass index (BMI) (29.5 ± 6.8 vs. 27.5 ± 6.3 kg/m^2^, Wilcoxon rank-sum *P* = 0.08, adjusted *P* = 0.07). Statistical models that additionally adjusted for the other drugs under consideration yielded very similar results: there was no change in statistical significance for any comparison except that HDL cholesterol concentrations were significantly higher in patients taking antimalarials (*P* = 0.03).

## Discussion

The major findings of this study are that in a cross-sectional setting drugs used to treat RA did not have major adverse effects on cardiovascular risk factors and antimalarial use was associated with beneficial lipid profiles.

### Corticosteroids

Corticosteroids are thought to induce dyslipidemia through impaired catabolism of LDL, and increased lipoprotein lipase activity [17]. They can also induce hypertension and glucose intolerance [8,17]. Although we found that corticosteroid use was associated with increased HDL concentrations, this requires cautious interpretation in terms of implications for cardiovascular risk, since a drug that raised HDL cholesterol was associated with increased mortality in randomized trials [18,19] and the functional capacity of HDL may be more important than its concentrations. Our findings in RA differ from those in SLE where corticosteroid use was associated with increased triglycerides [20]. The reason for this difference is not clear, but may be related to the more frequent use of high doses of corticosteroids in SLE.

### Methotrexate

The reported association between methotrexate use and increased concentrations of homocysteine, a cardiovascular risk factor, is a concern [21]. We found that homocysteine concentrations did not differ in patients receiving or not receiving methotrexate; this may have been the result of concurrent folate administration [22], which is common practice and routine in our cohort.

### Antimalarials

Antimalarials may have potential benefits on cardiovascular risk since they are associated with lower LDL cholesterol and triglycerides, concordant with other studies [9,23]. They were also associated with lower blood pressure. While the hypotensive effects of antimalarials are well known [24,25], these findings were mainly with chloroquine used to treat malaria and not with hydroxychloroquine in a rheumatological setting. Thus, our findings suggesting that hydroxychloroquine is associated with lower blood pressure in a rheumatological setting, is a novel finding. The effects of antimalarials in this cohort of patients with RA are much more prominent than in our previous report in patients with SLE in whom use of antimalarials was not associated with differences in cardiovascular risk factors [20].

### NSAIDs and COX-2 Inhibitors

The lack of a significant association between the use of NSAIDs and COX-2 inhibitors and cardiovascular risk factors in this RA cohort replicates findings in SLE [20] but needs cautious interpretation; the adverse effects of NSAIDs and COX-2 inhibitors, particularly on blood pressure, are well documented [5–7]. There could be a bias by indication to select low-risk patients for such therapies. Also, patients who had previously become hypertensive on NSAID therapy may have had these medications discontinued.

### Leflunomide and TNF-α Blockers

Associations between leflunomide and cardiovascular risk factors are not well described although there are some reports of leflunomide increasing blood pressure [26]. The finding of lower plasma glucose concentrations with leflunomide use is novel and may derive in part from a decrease in body weight, a known side-effect of leflunomide [27]. TNF-α blockers, may improve insulin sensitivity [28], but not lipid concentrations [29]; in our study no effects on glucose or lipid concentrations were observed.

### Limitations of the Study

This study has several limitations. First, it was cross-sectional, and the pattern of drug use may have been affected by indication bias. Also current drug use does not always reflect the true effects of the drug since the duration of drug use needs to be considered, which was not available; we did not analyze information about past use of a drug in patients not currently taking a particular drug. Also, we did not study additional cardiovascular risk markers such as EKG findings or elevated BNP levels.

Second, since the study was exploratory, we performed multiple statistical comparisons without statistical adjustment and the findings should be interpreted in that light. Nevertheless, since randomized controlled trials to examine the effects of the drugs of interest on cardiovascular risk factors in RA are not feasible, the study provides useful information, despite its limitations.
